# Molecular Epidemiology and Comparative Genome Analysis of Clinically-isolated MRSA Strains in Indonesia

**DOI:** 10.14789/jmj.JMJ21-0040-OA

**Published:** 2022-04-20

**Authors:** FIRMAN SETIAWAN, TADASHI BABA, YUH MORIMOTO, YOSUKE NAKAGAWA, RYOTARO SHIMOGAMI, TERUO KIRIKAE, KEIICHI HIRAMATSU

**Affiliations:** 1 Department of Neurology, Juntendo University School of Medicine, Tokyo, Japan; 1 Department of Neurology, Juntendo University School of Medicine, Tokyo, Japan; 2 Department of Medical Microbiology, Airlangga University Faculty of Medicine, Jawa Timur, Indonesia; 2 Department of Medical Microbiology, Airlangga University Faculty of Medicine, Jawa Timur, Indonesia; 3 Department of Bacteriology, Juntendo University Graduate School of Medicine, Tokyo, Japan; 3 Department of Bacteriology, Juntendo University Graduate School of Medicine, Tokyo, Japan; 4 Graduate School of Nursing, Seisen Jogakuin College, Nagano, Japan; 4 Graduate School of Nursing, Seisen Jogakuin College, Nagano, Japan; 5 Faculty of Health Science, Juntendo University, Tokyo, Japan; 5 Faculty of Health Science, Juntendo University, Tokyo, Japan; 6 Department of Bacteriology, Juntendo University, Tokyo, Japan; 6 Department of Bacteriology, Juntendo University, Tokyo, Japan; 7 Department of Infection Control Science, Juntendo University School of Medicine, Tokyo, Japan; 7 Department of Infection Control Science, Juntendo University School of Medicine, Tokyo, Japan

**Keywords:** Methicillin-resistance *Staphylococcus aureus* (MRSA), Staphylococcal cassette chromosome mec (SCC*mec*), Indonesia, Complete genome determination

## Abstract

**Objective:**

Most strains of methicillin-resistant *Staphylococcus aureus* (MRSA) analyzed to date have been from industrialized countries, with information lacking on the epidemiology of MRSA in other regions of the world. The present study describes the molecular epidemiology of MRSA strains collected at a referral hospital in Surabaya City, Indonesia in 2015-2016. The similarity of strains isolated in Indonesia to known lineages of MRSA was investigated.

**Materials:**

Of 45 MRSA strains isolated in Surabaya, 10 were selected by antibiotic resistance patterns and clinical features, while excluding duplicates. Methods: Whole genome sequencing was performed using a next-generation sequencer, and the complete genome sequence of one of these 10 strains was also determined by the PacBio system. The strains were subjected to molecular epidemiological analyses, including the presence of drug-resistance and virulence-related genes, the determination of sequence types and staphylococcal cassette chromosome *mec* (SCC*mec*) types and mutual phylogenetic relationships, using standard analytical tools.

**Results:**

The molecular types of these MRSA strains showed significant diversity. Complete sequencing of the genome of strain IDSA1 showed that it belonged to the ST239 group, while also having unique mobile genetic elements. Conclusions: Despite the small number of MRSA strains collected in a limited area and over a short period of time, these strains were found to have arisen in many other regions of the world, suggesting that they had migrated into Indonesia through human movement. These strains also showed molecular differentiation after migrating into Indonesia.

## Introduction

*Staphylococcus aureus* is a microorganism known to cause hospital, community and livestock infections, as well as fatal diseases such as toxic shock, bacteremia, sepsis, necrotizing pneumonia and endocarditis. The emergence of drug-resistance strains of *S. aureus*, including multidrug-resistant *S. aureus* (MRSA) has become a worldwide concern, with MRSA infections causing approximately 20,000 deaths per year in the United States^1)^, and about 4,000 in Japan^2)^. Determination of the molecular epidemiology of MRSA strains has allowed prediction of their regional spread and the design of effective chemotherapeutic agents against these infectious diseases. Methods of molecular epidemiological analyses include multi-locus sequence types (MLST)^3)^; searches and typing of mobile genetic elements, drug-resistance genes and virulence-related genes; and phylogenetic analysis based on single nucleotide polymorphisms (SNPs). Molecular epidemiological analyses of MRSA have also included typing of staphylococcal protein A genes based on their polymorphism^4)^ and typing of the staphylococcal cassette chromosome *mec* (SCC*mec*), a determinant of resistance to *β*-lactam antibiotics^5)^.

The results of molecular analyses are available as databases listed on websites. One example is PubMLST (https://pubmlst.org)^6)^, a collection of MLST of various microorganisms. The webpage shows lists of bacterial strains and their MLST by countries. Several hundred strains of *S. aureus* have been identified to date in industrialized countries, with much less information available about *S. aureus* strains in other regions of the world. For example, as of July 2021 no strain of *S. aureus* had been isolated in the Republic of Indonesia, although this country has a population of 264 million residents, the fourth highest in the world in 2018, and a gross domestic product based on purchasing power parity of 3.9 trillion dollars, the seventh highest in the world in 2018. Due to its large population and economic potential, Indonesia can play an important role in the appearance and spread of new molecular types of MRSA strains, similar to other industrialized countries. Therefore, the molecular epidemiological analyses of MRSA strains isolated in Indonesia are needed to predict the appearance of MRSA strains.

The present study analyzed MRSA strains obtained from the clinical microbiology laboratory at a leading referral hospital in Surabaya City, Jawa Timur, Indonesia. Of these strains, 10 were subjected to whole genome sequencing by employing a next generation sequencer, with analyses showing that these strains varied in sequence types, as defined by MLST, the presence of mobile genetic elements including SCC*mec*, virulence factors and drug resistance genes. The results strongly suggested that these strains had originated in various regions and from various sources throughout the world. Furthermore, complete genome sequencing of one of these strains, followed by detailed comparative analyses with a known *S. aureus* genome, showed that the genome of this MRSA strain underwent rapid alteration after migration into Indonesia.

## Materials and methods

**Statement on ethics control and appropriateness of the experiments.** All of the methods and the experimental protocols employed in this study were performed in accordance with relevant guidelines and regulations and were approved by Juntendo University School of Medicine Research Ethics Committee (permission #2019041). Informed consent was obtained from all participants. Prior to starting this study, all participating researchers had completed an ethics training course provided by Association for the Promotion of Research Integrity, Tokyo, Japan.

**Bacterial isolates and patient characteristics.** Single specimens were obtained from 121 patients and grown on mannitol salt agar. Each of these specimens yielded a single strain with a yellowish pigment and background, suggesting that they were strains of *S. aureus*. These strains were subsequently isolated on tryptic soy agar as single colonies. Forty-five of these strains, designated Infectious Diseases Society of America (IDSA) strains, were subjected to Microflex Biotyper matrix-assisted laser desorption ionization/time of flight mass spectrometry (MALDI-TOF MS)^7)^ and identified by comparison with a database complete as of March 2018 (Bruker, Billerica, MA, USA). Ten of these 45 MRSA strains were selected after categorizing them by patterns of antibiotic resistance and clinical features, while omitting strains with the same features. Strain identification was confirmed by sequencing of the 16S ribosomal RNA genes as part of whole genome sequencing.

**DNA manipulation, genome sequencing, annotation, species identification and comparisons with other *S. aureus* strains.** The genomic DNAs of *S. aureus* strains used in this study were extracted and purified using ISOPLANT II kits (NipponGene, Tokyo, Japan). After preparing libraries with Nextera XT library preparation kits (Illumina), the genomic DNAs were subjected to whole-genome sequencing using a MiSeq Next generation sequencer (Illumina, Inc. San Diego, CA, USA). The genome of IDSA1 was also subjected to PacBio RS II (Pacific Biosciences, Menlo Park, CA, USA) to determine the complete genome. Total reads of 883.6 Mbp (285x coverage) were assembled with HGAP 3.0^8)^. Following complete genome sequencing, the 16S ribosomal RNA gene sequence of IDSA1 was compared with identical sequences in the databases. Average nucleotide identity (ANI) analysis was performed^9, 10)^ using an ANI calculator^11)^ on the EZbiocloud homepage (https://www.ezbiocloud.net/tools/ani), which confirmed that the IDSA strains were *S. aureus*. Coding sequences (CDS) and ribosomal and transfer RNA genes were extracted, along with their initial functional assignments, using the RASTtk algorithm^12)^ available on the RAST annotation server^13)^. The CDS annotations were confirmed by visual comparisons with known gene products on *In Silico* Molecular Cloning (IMC) software (In Silico Biology, Inc., Kanagawa, Japan), which assists in evaluating the prevalence of the annotated sequence by comparison of each CDS with its homologues registered in databases. IMC software was also used for circular genome display and comparative analyses of the IDSA1 genome with the genome of *S. aureus* strain TW20. The sequence and annotation data of IDSA1 have been registered in the databases under accession number AP025249 .

**Determination of minimum inhibitory concentrations of IDSA strains against antibiotics.** Minimum inhibitory concentrations (MICs) of the MRSA strains against various antimicrobial reagents were determined by the microbroth dilution method according to Clinical and Laboratory Standards Institute (CLSI) guidelines^14)^.

**Computer-based molecular epidemiology and other genome analyses.** MLST^3)^ was determined by depositing the whole genome sequences of IDSA strains in the Center for Genomic Epidemiology (CGE) website (http://www.genomicepidemiology.org). *S. aureus* virulence genes were identified using VirulenceFinder 2.0^15)^ of the CGE server with thresholds of 90% nucleotide sequence identity and 60% minimum length. Drug resistance genes were initially identified by ResFinder 3.2^16, 17)^ on the CGE server, followed by one-to-one visual inspection of annotated genes. The phylogenetic relationships among sequenced *S. aureus* strains were analyzed by CSI Phylogeny 1.4^18)^ on the CGE server, a method based on single nucleotide polymorphisms (SNPs) among genomes, allowing a maximum-likelihood phylogenetic tree to be drawn^18)^. Based on the results of analysis of CSI phylogeny, and using a NEWICK-format file, a tree was re-drawn as a radial layout with a root centered by FigTree ver. 1.4.4 software (http://tree.bio.ed.ac.uk/, kindly gifted by Professor Andrew Rambaut of the Institute of Evolutionary Biology at the University of Edinburgh). The IMC software described above was used to assess GC contents, GC-skewing and dot plots to identify homologous regions in two genomes. Types of SCCmec were determined by SCCmecFinder 1.2^19, 20)^ on the CGE server.

## Results

**Drug resistance, virulence and other genetic features of the IDSA strains**. Analysis of the MICs of the 10 selected IDSA strains showed that these strains were multidrug-resistant (Table 1). Whole genome sequencing with a next-generation sequencer showed that these strains consisted of several tens of contigs, allowing molecular epidemiological analyses. Sequencing of their 16S ribosomal genes and determination of their average nucleotide identity (ANI) showed that all of these strains were *S. aureus*. These strains varied in their drug resistance determinants (Table 2). The presence of genes confirming resistance to *β*-lactams and aminoglycosides was consistent with the determined MICs tested; whereas others were not (Table 1). This may have been due to the involvement of functionally-uncharacterized genes that confer antibiotic resistance to these strains.

**Table 1:** Minimum inhibitory concentrations of the MRSA strains analyzed in this study. Numbers are in *μ*g/ml

strains	MPIPC^a)^	ABPC^b)^	CEZ^c)^	CMZ^d)^	FMOX^e)^	IPM^f)^	GM^g)^	ABK^h)^	MINO^i)^	CFX^j)^	EM^k)^	CLDM^l)^	VCM^m)^	TEIC^n)^	LZD^o)^	FOM^p)^	ST^q)^	LVFX^r)^
IDSA1	R^s)^	R	>16	>32	>16	>8	>8	4	≤2	>16	>4	2	2	2	2	≤32	>38/2	R
IDSA2	R	R	>16	>32	>16	>8	>8	4	≤2	>16	>4	>2	≤0.5	1	2	≤32	>38/2	R
IDSA3	R	R	4	8	4	0.5	>8	1	≤2	16	0.5	0.25	1	≤0.5	2	≤32	>38/2	R
IDSA5	R	R	>16	>32	>16	>8	>8	2	8	>16	0.5	0.25	1	1	4	≤32	≤9.5/0.5	R
IDSA6	R	R	8	8	4	0.5	>8	0.5	≤2	16	>4	2	1	≤0.5	2	≤32	≤9.5/0.5	R
IDSA8	R	R	>16	8	8	2	0.5	1	≤2	>16	0.5	0.25	1	≤0.5	4	≤32	≤9.5/0.5	R
IDSA 13	R	R	4	8	2	≤0.25	>8	1	≤2	16	0.5	0.12	1	≤0.5	2	≤32	≤9.5/0.5	R
IDSA 17	R	R	2	8	2	≤0.25	>8	0.5	≤2	16	0.5	0.25	1	≤0.5	2	<32	>38/2	R
IDSA 20	R	R	4	8	2	≤0.25	>8	2	≤2	>16	0.5	0.12	1	1	2	≤32	≤9.5/0.5	R
IDSA 44	R	R	16	8	4	≤0.25	0.5	0.5	≤2	>16	2	0.25	1	≤0.5	4	≤32	≤9.5/0.5	R

(a) oxacillin, (b) ampicillin, (c) cefazolin, (d) cefmetazole, (e) flomoxef, (f) imipenem, (g) gentamicin, (h) arbekacin, (i) minocycline, (j) cefoxitin, (k) erythromycin (l) clindamycin, (m) vancomycin, (n) teicoplanin, (o) linezolid, (p) fosfomycin (q)sulfamethoxazole/trimethoprim, (r) levofloxacin (s) resistance.

**Table 2:** Drug resistance genes found in the MRSA strains analyzed in this study

strains	SCC*mec*Type	ST^(a^	b-lactam	aminoglycoside	tetracycline	macroride	trimethoprim	quinolone	fucidic acid
IDSA1	typeIII(3A)	ST239	*blaZ, mecA*	*aac(6')-aph(2"), aph(3')-III , ant(9)-Ia*	*tetM*	*ermA*	*dfrG*	*gyrA*^(b^, *parC*^(b^	*fusA* ^(b^
IDSA2	typeV(5C2&5)	ST97	*blaZ, mecA*	*aac(6')-aph(2")*					*fusC*
IDSA3	typeV(5C2&5)	ST789	*blaZ, mecA*	*aac(6')-aph(2"), aph(3')-III*	*tetK*		*dfrG*	*gyrA, parC*	
IDSA5	typeIII(3A)	ST239	*blaZ, mecA*	*ant(6)-Ia, aph(3')-III*	*tetK, tetM*				
IDSA6	typeV(5C2&5)	ST789	*mecA*	*aac(6')-aph(2")*			*dfrG*	*gyrA, parC*	
IDSA8	typeIVd(2B)	ST672	*blaZ, mecA*				*dfrB(b*	*gyrA, parC*	
IDSA 13	typeV(5C2&5)	ST789	*blaZ, mecA*	*aph(3')-III*	*tetK*		*dfrG*	*gyrA, parC*	
IDSA 17	typeV(5C2&5)	ST789	*blaZ, mecA*	*aac(6')-aph(2"), aph(3')-III*	*tetK*		*dfrG*	*gyrA, parC*	
IDSA 20	typeV(5C2&5)	ST789	*blaZ, mecA*	*aac(6')-aph(2")*				*gyrA, parC*	*fusC*
IDSA 44	typeVc(5C2&5)	ST30	*mecA*		*tetK*			*gyrA, parC*	

(a) Sequence type. (b) *gyrA, parC, fusA* and *dfrB* genes are not resistance genes per se. Point mutations in these genes confer resistance.

**Figure 1:**
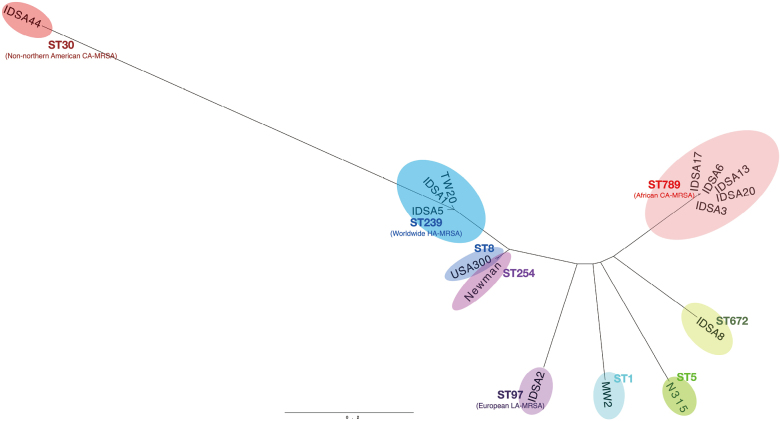
Whole genome-wide phylogenetic relationship among S. aureus strains isolated at a referral hospital in Surabaya City, Indonesia, during 2015-2016 Maximum-likelihood tree based on the mapping of SNPs in the genomes to the reference sequence of *S. aureus* strain USA300^33)^. The branch length indicates the proportions of SNPs relative to the total 21,646 SNPs in the 14 genomes (refer to the scale bar). The whole genome sequences of the *S. aureus* strains available in databases and the genomes of the N315^27)^, MW2^24)^ and IDSA strains (this study) were subjected to CSI Phylogeny 1.4^18)^ using default parameters (minimum depth at SNP positions: 10, relative depth at SNP positions: 10, minimum distance between SNPs: 10, minimum SNP quality: 30, minimum read mapping quality: 25, minimum Z-score: 1.96 while ignoring heterozygous SNPs). The sequence types (ST) of the IDSA strains, determined based on MLST analysis^3)^, are indicated with colored ovals, as are the typical regions and sources of the STs. HA-MRSA, hospital-acquired MRSA; CA-MRSA, community-acquired MRSA; LA-MRSA, livestock-associated MRSA. ST672 is a very rare clone, with only 18 reports; it has been isolated in Australia, India, Iran, Haiti and the USA according to the records in PubMLST^6)^ as of August 25, 2021.

The strains also showed variations in their possessions of virulence-related genes (Table 3). Variations in drug resistance genes as well as in virulence factors do not always reflect whole genome-wide similarities, because drug-resistance and virulence-related genes are often encoded on mobile genetic elements and may therefore migrate among strains, in contrast to their chromosomes. These findings could not exclude the possibility that the MRSA strains were closely related based on their genetic background. The whole-genome wide diversities of the MRSA strains were assessed by analyzing their MLST. This analysis allows bacterial strains to be categorized by their genetic lineages, thus determining their phylogenetic relationship. The MLST of the MRSA strains used in this study are summarized in Table 2. This analysis showed 11 strains belonging to as many as five sequence types (ST), indicating marked diversity in their genetic backgrounds. Because MLST analysis shows diversities in limited numbers of housekeeping genes, phylogenetic analyses were performed based on SNPs that covered the entire genome. These analyses revealed large variations in the genomes of these strains, regardless of regional and periodic limitations of the MRSA isolation (Figure 1). These results strongly suggest that these MRSA strains were derived from various parts of the world.

**Table 3:** Virulence determinants found in the MRSA strains analyzed in this study

strains	exoenzymes	blood cell lysins	Superantigens	hostimm^(a^
IDSA1	*splA, splB, splE, aur*	*hlg, lukDE*	*sea*	*sak, scn*
IDSA2	*splA, splB, splE, aur*	*hlg*		*sak, scn*
IDSA3	*splA, splB, splE, aur*	*hlg, lukDE*	*sep*	*sak, scn*
IDSA5	*splA, splE, aur*	*hlg*	*sea*	*sak*
IDSA6	*splA, splB, splE, aur*	*hlg, lukDE*	*sep*	*sak, scn*
IDSA8	*aur*	*hlg, lukDE*	*seg, sem, sen, seo, seu*	*sak, scn*
IDSA 13	*splA, splB, splE, aur*	*hlg, lukDE*	*sep*	*sak*
IDSA 17	*splA, splB, splE, aur*	*hlg, lukDE*	*sep*	*sak*
IDSA 20	*splA, splE, aur*	*hlg, lukDE*		
IDSA 44	*splE*	*hlg, lukFS-PV*	*seg, sei, sem, sen, seo, seu*	*sak, scn*

(a) Host-immune system modulators.

**Overview of the IDSA1 genome and comparison of *S. aureus* strain TW20 with a similar genetic background.** The analyses above showed that three strains belonged to ST239. Because these strains are resistant not only to penicillin but to cephem, the spread of this lineage could become a public concern in Indonesia. To determine the molecular bases of this lineage, one of these ST239 MRSA strains, IDSA1, was subjected to complete genome determination. The genome of IDSA1 consisted of a single chromosome of 3,096,213 base pairs, with no plasmids. The genome of strain GW20 was highly similar to that of IDSA1, with both classified as ST239 and type-III SCC*mec*. Despite their common features^21)^, the IDSA1 genome was longer in size than the TW20 genome, which consists of 3,043,210 base pairs, because IDSA1 possesses two more prophages than TW20 (Table 4). ST239 is a hospital-acquired MRSA clone^22)^, observed throughout the world. It is noteworthy that this clone was also found in Indonesia, which is geographically isolated from continents.

**Table 4:** Complete genome statistics of IDSA1 and TW20

strain		IDSA1	TW20
Length of sequence (bp)		3096213	3043210
G+C contents (%)		32.81	32.78
No. of protein coding regions		2971	2941
No. of rRNAs	5S	6	6
	16S	5	5
	23S	5	5
No. of tRNAs		60	60
No. of prophages		5	3

**Figure 2:**
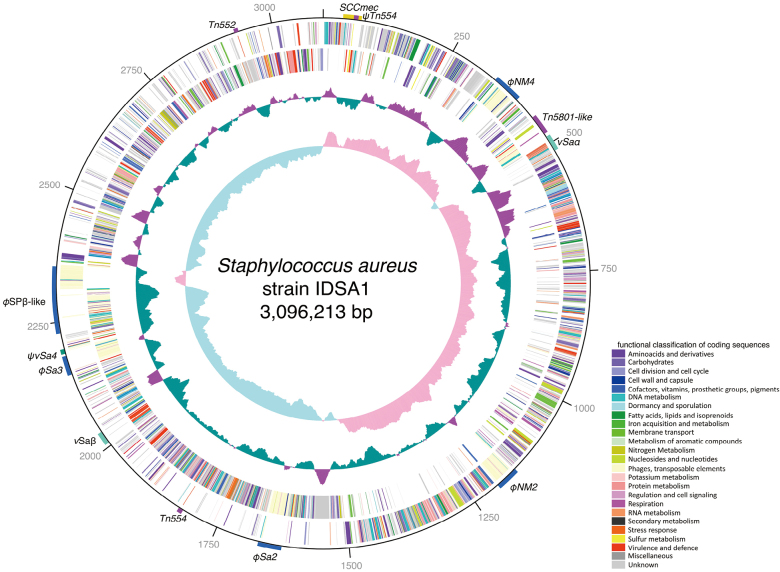
Functional genomic organization of the chromosome of *Staphylococcus aureus* strain IDSA1 The first outermost circle is scale in kilobase pairs. The second and third circles show open reading frames on the plus and minus strands, respectively. The fourth circle shows G+C contents, with purple indicating higher than average; and the fifth circle shows GC-skew, with pink indicating higher than average. The positions of mobile genetic elements, including *SCCmec*, prophages, transposons and other genomic islands, are indicated outside the first circle (see also Figure 3).

**Figure 3:**
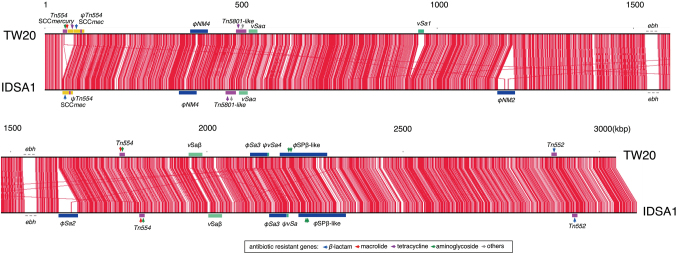
Genome rearrangement map of *Staphylococcus aureus* strain IDSA1 compared with *S. aureus* strain TW20 Regions of >90% nucleotide identity are shown with red lines, illuminating gaps representing regions specific to each strain. Positions of mobile genetic elements are indicated as in Figure 2.

Figure 2 shows the IDSA1 chromosome with the functional categories of each gene. Its GC skew values corresponded to the tendency of gene orientation, as typically seen in *S. aureus* genomes^23)^. The positions of mobile genetic elements, including prophages, transposons, SCC and other genomic islands^24)^, are highlighted. Figure 3 is a global genome rearrangement map between IDSA1 and TW20 that allows comprehensive visualization of insertions and deletions. The red lines show the start positions of common coding sequences (CDS) on both chromosomes. The extraordinarily large CDS seem like gaps in principle, despite the two genomes sharing the CDS. The gap appearing approximately at the 1.5 Mbp position is such an example, corresponding to *ebh* genes^24)^, which code for proteins of over 10,000 amino-acids. The mobile genetic elements are highlighted in Figures 2 and 3, and the positions of drug resistance genes are indicated in Figure 3. Many drug resistance genes were found to be accompanied by mobile genetic elements. For example, the *blaZ* gene, which encodes the *β*-lactam degrading enzyme BlaZ, was found to be present on transposon Tn*552* on both chromosomes, together with the sensory and regulatory genes *blaR* and *blaI*, respectively^25)^, although blaZ, blaR and blaI genes are found on plasmids in many other *S. aureus* strains^26)^. The IDSA1 and TW20 strains also shared Tn*5801*-like elements with the *tetM* (tetracycline-resistant determinant) and *dfrG* (trimethoprim-resistant determinant) genes^27, 28)^, Tn*554* with *tetM* (etracycline-resistant determinant)^27)^ and *ant*(*9*)-*Ia* (aminoglycoside- resistant determinant), *φ*SPb-like prophages with *aac*(*6’*)-*aph*(*2”*) and *aph*(*3’*)-*III* (aminoglycoside- resistant determinants) and Type-III SCC*mec*s with *mecA* (penicillin-binding protein with lower affinity to b-lactams, conferring bacterial resistance to these reagents)^21)^.

Although the IDSA1 and TW20 strains had the same ST, other mobile genetic elements differed in IDSA1 and TW20. For example, IDSA1 possessed, two prophages *φ*NM2^29)^ and *φ*Sa2^24)^, which were absent from TW20. These two prophages did not encode any known drug-resistance or virulence-related genes. In contrast, IDSA1 lacked the *ν*Sa1 element^30, 31)^, which frequently carries genes that encode enterotoxins K and Q. IDSA1 also lacks SCC*mercury*^32)^, which carries mercury resistance genes, an SCC that tends to be tandemly inserted with SCC*mec*. Although some positions of transposons and insertion sequences were similar in the two chromosomes, others were different. For example, two Tn*554* and a pseudo Tn*554* (*ψ*Tn*554*) in IDSA1 were found to be inserted into the corresponding positions of TW20, whereas TW20 has an extra Tn*554* carrying the genes *ant*(*9*)-*Ia* (an aminoglycoside resistant determinant) *ermA* (a macrolide resistant determinant) adjacent to SCC*mercury*. The two chromosomes shared eight IS256, however, IDSA1 had eight additional IS256 (data not shown).

## Discussion

To our knowledge, this is the first intensive study of the molecular epidemiology of MRSA strains isolated in Indonesia. Whole genome sequences of these MRSA strains, which were isolated from patients in a hospital in Surabaya City, Indonesia, over a one-year period, differed in their genetic backgrounds, suggesting that they had originated from many regions of the world. These strains includeed lineages of ST30 (typically seen in community-acquired MRSA [CA-MRSA] isolated worldwide, except for northern America), ST239 (typically seen in worldwide hospital-acquired MRSA [HA-MRSA]) ST97 (typically seen in European livestock-associated MRSA [LA-MRSA]), and ST789 (typically seen in African CA-MRSA)^22)^. Also isolated was ST672, information on which has been very limited to date. These findings suggest that MRSA strains migrate with human movement. Indeed, many Indonesian people go to other countries as migrant laborers, which may result in their acquisition and spread of MRSA strains with various genetic backgrounds throughout the country. This possibility is of great concern in determining effective chemotherapy and in reducing the risk of exposure to antimicrobial resistance microbes; as it can result in the regional spread of MRSA strains with resistance to multiple antimicrobial agents. This, in turn, could increase the ingestion of multiple types of antimicrobials, subsequently leading to more frequent appearance of drug-resistance microbes. Because MICs have been determined for very few strains of MRSA isolated from patients in Indonesia, it is difficult to determine the proper treatment of each infection.

Of the IDSA strains isolated, one, IDSA1, which belongs to the ST239 lineage, was subjected to whole genome sequencing. This may be the first report describing the complete genome sequence of an MRSA strain isolated in Indonesia. Comparative genome analyses with the *S. aureus* strain TW20 showed that both this strain and IDSA1 were of the same ST and SCC*mec* types. They differed in possession of some mobile genetic elements that affected the presence or absence of some drug-resistance and virulence related determinants. Mobile genetic elements may be acquired or lost more rapidly than migration of strains with a specific ST to another geographical region. This hypothesis can be tested by acquiring more information on the regional molecular epidemiology of MRSA strains. Such a study could contribute to the determination of trends of MRSA strains.

## Patient Consent for Publication Statement

Clinical isolates taken during routine clinical microbiology laboratory examination and human carriage swabs were provided voluntarily by patients in Surabaya, Indonesia, in 2015, with all patients providing written informed consent.

## Funding

This work was primarily supported by a Grant-in-aid for special research in subsidies for ordinary expenses of private schools from Promotion and Mutual Aid Corporation for Private Schools of Japan. This work was also supported by grants from the Research Program on Emerging and Re- emerging Infectious Diseases from Japan Agency for Medical Research and Development (Grant number 19fk0108061h0302).

## Author contributions

All authors contributed to the study conception and design. Strain and DNA manipulation and MIC determination: FS and YM; Funding acquisition: TK and KH; Writing, reviewing and editing the manuscript: FS and TB; In silico genome analyses and preparation and drawing figures: TB, YN and RS. Supervision and project administration: TB. All authors read and approved the final manuscript, except for KH who passed away on the 5th of June, 2020. KH had approved the draft version of this manuscript before passing.

## Conflicts of interest statement

The authors declare that they have no conflicts of interest.
